# The Endocannabinoid System: Implications in Gastrointestinal Physiology and Pathology

**DOI:** 10.3390/ijms26031306

**Published:** 2025-02-03

**Authors:** Emanuela Aloisio Caruso, Valentina De Nunzio, Valeria Tutino, Maria Notarnicola

**Affiliations:** 1 Laboratory of Nutritional Biochemistry, National Institute of Gastroenterology IRCCS “Saverio de Bellis”, 70013 Castellana Grotte, Bari, Italy; emanuela.caruso@irccsdebellis.it (E.A.C.); valentina.denunzio@irccsdebellis.it (V.D.N.); 2 Laboratory of Clinical Pathology, National Institute of Gastroenterology IRCCS “Saverio de Bellis”, 70013 Castellana Grotte, Bari, Italy; valeria.tutino@irccsdebellis.it

**Keywords:** endocannabinoid system, gastrointestinal physiology, obesity, non-alcoholic fatty liver disease, irritable bowel syndrome, colorectal cancer

## Abstract

The endocannabinoid system (ECS), composed of receptors, endocannabinoids, and enzymes that regulate biosynthesis and degradation, plays a fundamental role in the physiology and pathology of the gastrointestinal tract, particularly in the small and large intestine and liver. Specifically, cannabinoid receptor type 1 (CB1R) and cannabinoid receptor type 2 (CB2R), located principally in the nervous system and immune cells, orchestrate processes such as intestinal motility, intestinal and hepatic inflammation, and energy metabolism, respectively. The main endocannabinoids, anandamide (AEA) and 2-arachidonoylglycerol (2-AG), influence appetite, body weight regulation, and inflammatory states and thus have implications in obesity, non-alcoholic fatty liver disease (NAFLD) and irritable bowel syndrome (IBS). Recent studies have highlighted the therapeutic potential of targeting the ECS to modulate gastrointestinal and metabolic diseases. In particular, peripheral CB1R antagonists and CB2R agonists have shown efficacy in treating intestinal inflammation, reducing hepatic steatosis, and controlling IBS symptoms. Moreover, the ECS is emerging as a potential target for the treatment of colorectal cancer, acting on cell proliferation and apoptosis. This review highlights the opportunity to exploit the endocannabinoid system in the search for innovative therapeutic strategies, emphasizing the importance of a targeted approach to optimize treatment efficacy and minimize side effects.

## 1. The Endocannabinoid System (ECS)

In 1988, Howlett and Devane discovered a complex receptor system, which they named the endocannabinoid system (ECS) [1].

The discovery of this system began with the development, around the 1960s, of trans-Δ^9^-tetrahydrocannabinol (Δ^9^-THC)-based drugs [2].

The proteins included in this system belong to three categories: the receptors, ligands, and enzymes responsible for their biosynthesis and degradation.

### 1.1. Endocannabinoids (eCBs)

The term “cannabinoids” refers to compounds present in the plant Cannabis Sativa, one of the first plants cultivated by humans, which has been used as a medicine since ancient times [2].

The main cannabinoids are Δ^9^-tetrahydrocannabinol (Δ^9^-THC), the component most commonly found in cannabis and responsible for its psychoactive properties, and cannabidiol (CBD), the second highest component in abundance, which has no psychoactive effects. Following the discovery of the receptors of these substances, endogenous substances that could act as ligands and activate the receptors were investigated. Endogenous cannabinoid receptor agonists were then classified as “endocannabinoids” (eCBs).

The class of endocannabinoids includes *N*-arachidonoylethanolamine (anandamide, AEA), 2-arachidonoylglycerol (2-AG), 2-arachidonylglyceryl ether (noladin ether), *N*-arachidonoyldopamine (NADA), and O-arachidonoylethanolamine (virodhammine, O-AEA) [3]. eCBs are lipid mediators derived from long-chain polyunsaturated fatty acids, which include amides, esters, and ethers, and mimic the action of THC. The best-known eCBs are anandamide, *N*-arachidonoylethanolamine (AEA), and 2-arachidonoylglycerol (2-AG) [4].

AEA was the first endocannabinoid identified in swine brains and is a member of the *N*-acylethanolamine (NAE) family [5], while 2-AG is a monoacylglycerol and was first isolated from rat brains and canine intestines [6]. Unlike classical peptide neurotransmitters, which are typically stored in vesicles and released in response to stimuli, endocannabinoids are synthesized on demand from lipid precursors in membranes [7].

**Figure 1:**
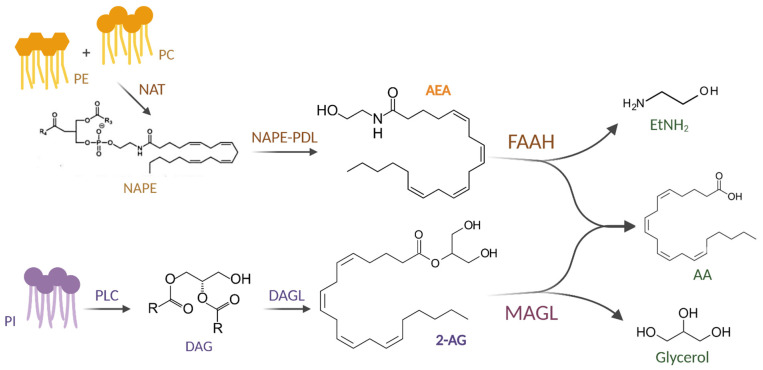
Diagram showing the biosynthesis and degradation of the two main endocannabinoids, anandamide and 2-arachidonoylglycerol. Abbreviations: PE: phosphatidylethanolamine; PC: phosphatidylcholine; NAT: *N*-acyl-transferase; NAPE: *N*-acyl-phosphatidylethanolamine; NAPE-PDL: *N*-arachidonoyl-phosphatidylethanolamine phospholipase type D; AEA: *N*-arachidonoylethanolamine; FAAH: fatty acid amide hydrolase; AA: arachidonic acid; EtNH_2_: ethanolamine; PI: phosphatidylinositol; PLC: phospholipase C; DAG: diacylglycerol; DAGL: DAG lipase; 2-AG: 2-arachidonoylglycerol; MAGL: MAG lipase.

AEA is mostly synthesized from membrane lipids, such as phosphatidylethanolamine (PE) and phosphatidylcholine (PC), which, through *N*-acyl-transferase (NAT), are converted to *N*-acyl-phosphatidylethanolamine (NAPE), which, in turn, can generate AEA through *N*-arachidonoyl-phosphatidylethanolamine phospholipase type D (NAPE-PDL) in four possible biosynthetic pathways. AEA is then predominantly hydrolyzed by the fatty acid amide hydrolase (FAAH)-1, which has been shown to hydrolyze AEA into arachidonic acid (AA) and ethanolamine (EtNH_2_) (Figure 1).

2-AG, on the other hand, is mainly produced by the hydrolysis of phosphatidylinositol (PI) by phospholipase C (PLC) to generate diacylglycerol (DAG), which is then later converted to 2-AG by α or β DAG lipases (DAGL). 2-AG is rapidly hydrolyzed into fatty acids (FAs), such as AA, and glycerol by several enzymes, particularly serine hydrolase MAG lipase (MAGL) [8] (Figure 1).

A number of pharmacological and genetic tools have been developed and used to elucidate the mode of action of signal transduction downstream of these receptors. Five categories of cannabinoids have been identified: classical cannabinoids, bicyclic cannabinoids, indole-derived cannabinoids, eicosanoids, and inverse antagonists/agonists. These show different selectivity for the two receptors, which allows the identification of the downstream effects of such signaling [9].

### 1.2. Receptor System

Three receptor classes have been distinguished: G-protein-coupled receptors (GPGR), such as CB1R and CB2R; nuclear receptors, such as peroxisome proliferator-activated receptors (PPARs); and ion channels, such as transient receptor potential cation channel subfamily V member 1 (TRPV1).

In 1990, the first ECS receptor, CB1R, an “orphan” GPCR, was discovered [10], and then in 1993, the second endocannabinoid receptor, CB2R, located mainly on blood cells and immune tissues, was characterized [11].

**Figure 2:**
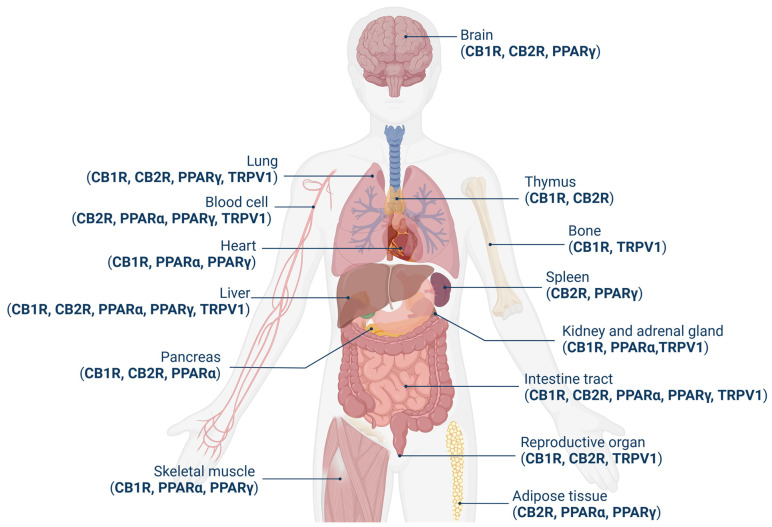
Graphical representation of anatomical distribution of cannabinoid receptors. Abbreviations: CB1R: cannabinoid receptor type 1; CB2R: cannabinoid receptor type 2; PPARα: peroxisome proliferator-activated receptor α; PPARγ: peroxisome proliferator-activated receptor γ; TRPV1: transient receptor potential cation channel subfamily V member 1.

The tissue localization of these receptors appears to be different (Figure 2). CB1R is one of the most abundant GPCRs found so far in the central nervous system (CNS) and reaches the highest density in the basal ganglia, cerebellum, hippocampus, and cortex, but it is also present in the peripheral nervous system (PNS) and several peripheral organs [12,13]. Its distribution is correlated with the observed effects of cannabinoids, including impairments in cognition, memory, learning, and motor coordination. Subsequent to its identification, CB1R has been found in a variety of circulating immune cells [14]. Their expression, although in smaller amounts, of CB1R has also been found at the peripheral level, particularly in the adrenal gland, heart, lung, prostate, liver, uterus, ovary, testis, vas deferens, bone marrow, thymus, and tonsils [14]. Of particular interest in the present context is the expression of CB1R in hepatocytes, hepatic sinusoidal cells, and stellate cells; even though this occurs at much lower concentrations than in the brain, it remains functionally relevant [15,16].

By contrast, CB2R is highly expressed in peripheral cells and organs with immune functions, including macrophages, B cells, natural killer cells, spleen, tonsils, thymus, and leukocytes, as well as in the gastrointestinal tract, lungs, and reproductive organ [14,17]; it was later found in several brain areas as well [9]. CB2R has also been detected in the liver but is predominantly expressed in Kupffer cells and stellate cells, albeit at low levels [18].

Several studies confirm the immune role of CB2R; the activation of this receptor by cannabinoids can, in fact, either suppress or enhance the production of pro-inflammatory agents, depending on the nature of the pro-inflammatory stimulus and the specific type of cannabinoid used [19]. The hepatic expression of these receptors is altered in different pathological situations, such as NAFLD [20], hepatic fibrosis [21], or hepatocellular carcinoma [22].

Members of the PPAs family of nuclear hormone receptors are transcriptional factors that regulate lipid metabolism, mitochondrial biogenesis, and energy homeostasis but also regulate immune functions. Several studies show that cannabinoids can bind these receptors and modulate neuroinflammation and neurodegeneration [23,24].

By activating PPARs, endocannabinoids exert a variety of effects influencing physiological and pathological processes, such as lipid metabolism, energy balance, feeding behavior, neuroprotection, epilepsy, circadian rhythms, inflammation, addiction, and cognitive function [25]. It has also been demonstrated that AEA can activate PPARγ and thus stimulate downstream signaling [26].

PPARα is highly expressed in organs and tissues with a high rate of fatty acid catabolism for energy production, such as the liver, brown adipose tissue, endocrine tissues, gastrointestinal tract, heart muscle, and skeletal muscle. To a smaller extent, PPARα is also found in the kidneys, adrenal tissues, endothelial cells, and immune system cells [27,28]

PPARγ is most highly expressed in adipose tissue but has also been found in other organs and tissues, including the liver, skeletal muscle, spleen, heart, placenta, lung, ovary, brain, immune system, and retina [29].

Synthetic and plant-derived cannabinoids attenuate neuroinflammation and neurodegeneration in animal models of acute or chronic neurodegenerative disorders through the activation of cannabinoid receptors and the PPARγ pathway [28]. AEA activates both PPARα and PPARγ but has a higher affinity for PPARα [24]. 2-AG also binds PPARα but with a lower affinity than AEA. Both 2-AG and 2-AG ether, a non-hydrolyzable analog of 2-AG, have been shown to activate PPARγ, suppressing the expression of IL-2 [30].

TRP channels are a large class of ion channels distributed on the plasma membrane of numerous animal cells and are involved in transduction in response to various chemical and physical stimuli. It has been shown that channels of several subfamilies, namely vanilloid (TRPV1–4), ankyrin (TRPA1), and melastatin (TRPM8), can be modulated by cannabinoids; they have, therefore, been classified as cannabinoid ionotropic receptors. Among them, vanilloid TRPV channels, in particular TRVP1, are activated by AEA or natural compounds such as THC and CBD [31,32].

### 1.3. Signaling Mechanisms

**Figure 3:**
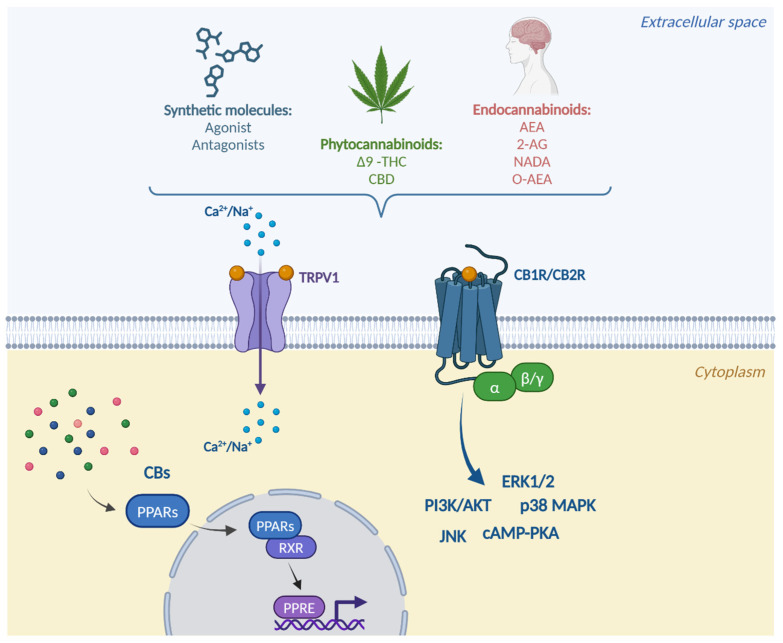
Molecular and signal transduction mechanisms downstream of CBs-receptor binding. Abbreviations: Δ^9^-THC: Δ^9^-tetrahydrocannabinol; CBD: cannabidiol; AEA: *N*-arachidonoylethanolamine; 2-AG: 2-arachidonoylglycerol; NADA: *N*-arachidonoyldopamine; O-AEA: O-arachidonoylethanolamine; TRPV1: transient receptor potential cation channel subfamily V member 1; CB1R: cannabinoid receptor type 1; CB2R: cannabinoid receptor type 2; CBs: cannabinoids; ERK: Extracellular signal-regulated kinases; MAPK: mitogen-activated protein kinase; PI3K: phosphoinositide 3-kinase; AKT: protein kinase B; JNK: c-Jun N-terminal kinase; cAMP: cyclic adenosine monophosphate; PKA: protein kinase A; PPARs: peroxisome proliferator-activated receptors; RXR: retinoid X receptor; PPREs: peroxisome proliferator response elements.

Downstream of cannabinoid-receptor binding, several molecular mechanisms occur that finally lead to signal transduction (Figure 3).

CB1R and CB2R belong to the class of GPCR and are essential for the regulation of human development and physiology. The signaling mechanisms include both G-protein-dependent and -independent processes. The binding of these receptors leads to the activation of Gi/o-protein-coupled receptors, which inhibits adenylate cyclase activity by reducing the intracellular accumulation of cyclic adenosine monophosphate (cAMP) [33].

The modulation of intracellular cAMP concentration by regulating the net phosphorylation of substrate proteins by protein kinase A (PKA) can cause important changes in cellular activity. Indeed, cAMP regulates the activity of many classes of ion channels, including voltage-dependent K^+^ and Ca^2+^ channels [34,35]. Therefore, at the neuronal level, these receptors have an effect on cell function and fate in general and, in particular, electrical activity and neurotransmitter release.

Another mechanism of action affects the immune response. CB1R and CB2R are expressed at the level of human lung macrophages and, when activated, induce the phosphorylation of extracellular signal-regulated kinases (ERK) 1 and 2 and the generation of reactive oxygen species (ROS). Furthermore, cannabinoid receptor activation selectively inhibits the release of angiogenic and lymphangiogenic factors, which may have a crucial effect on macrophage-assisted vascular remodeling in cancer, as well as chronic inflammation [36].

The stimulation of these receptors can also activate signaling pathways involving mitogen-activated kinases (MAPKs), including ERK1/2, p38 mitogen-activated protein kinases and c-Jun N-terminal kinase (JNK), but also the phosphoinositide 3-kinase (PI3K)/protein kinase B (Akt) pathway, which are associated with the regulation of cellular functions such as cell growth, transformation, and apoptosis [37]. Activated Akt phosphorylates intracellular substrates, promoting cell survival by inhibiting apoptosis through different targets, like Bad and caspase-9 [38].

The PPARs activated by cannabinoids are mostly PPARα and PPARδ. These are highly expressed in oxidative tissues and regulate genes involved in substrate transport, substrate oxidation, and oxidative phosphorylation [39]. As a result of the downstream mechanism of binding action and the activation of these receptors, after ligand binding, PPARs translocate into the nucleus, where they heterodimerize with the retinoid X receptor (RXR) and bind to peroxisome proliferator response elements (PPREs) in order to regulate the transcription of target genes [40].

The main TRP family ion channel activated by endocannabinoids is TRPV1. TRPV1, also known as the capsaicin receptor, is a cationic channel present in all major classes of nociceptive neurons. Upon the activation of this receptor, calcium moves through the pore, penetrates into the cell, and stimulates a series of calcium-dependent processes that lead to the desensitization of the channel, which cannot respond to further stimuli for a time, thus inducing the analgesic effect. CBD has also been shown to have this effect and, by desensitizing TRPV1, could have therapeutic potential against inflammatory and chronic pain [41].

A crosstalk mechanism has been established between the three receptor classes for parallel stimulation. A molecular link between CB1R and p53/miR-22/SIRT1/PPARα signaling in hepatocytes has also been demonstrated. Using bioinformatic techniques and in vitro and in vivo experiments, the authors found that hepatic CB1R increases the regulatory activity of p53 on miR-22, which, in turn, represses the expression and activity of PPARα and SIRT1, contributing to fat accumulation in the hepatocytes under hepatic steatosis conditions [42].

A molecular connection has also been demonstrated between CB1R and PPARγ in the abdominal adipose tissue of obese subjects. The stimulation of preadipocytes at the early stage of adipocyte differentiation with the non-selective cannabinoid agonist WIN55,212 induces a significant expression of PPARγ [43]. Another study showed that cannabinoid receptor activation with HU-210 stimulated PPARγ, an early marker of adipocyte differentiation, and inhibited the expression of adiponectin, which is a late marker of differentiation [44].

In a study using C2C12 cell cultures, mouse myoblasts, it was seen that trans-caryophyllene, a specific agonist of CB2R, can promote fatty acid oxidation through the peroxisome proliferator-activated receptor gamma 1α (PGC-1α) pathway. Activated CB2R stimulates sirtuin 1 (SIRT1) deacetylase activity by increasing the phosphorylation of the cAMP response element-binding (CREB) protein, leading to increased levels of PGC-1α deacetylation. PGC-1α, at the transcriptional level, increases the ability of the nuclear receptor PPARγ to promote the transcription of fatty acid oxidation enzymes and is upregulated by SIRT1 [45].

In another study, it was shown that in cultured rat microglia and human macrophages, the endogenous fatty acid amide palmitoylethanolamide (PEA) increases CB2R mRNA and protein expression through activating PPARα. This result was confirmed in several pharmacological, biochemical, and computational studies [46].

A further interesting cross-link was found between TRPV1 and PPARα. It was seen that in sensory neurons, in the presence of PEA and capsaicin, PPARα agonists, PPARα-dependent activation, and the desensitization of TRPV1 occur, triggering membrane depolarization and leading to a substantial increase in intracellular Ca^2+^ concentration. These results highlight the concept that the PEA-induced TRPV1 desensitization is a potential molecular mechanism underlying the analgesic actions of this acylethanolamide [47].

Baskaran et al. showed that capsaicin activating the TRPV1 channel allows Ca^2+^ accumulation in the cell, which increases SIRT1 expression and activity and triggers white adipose tissue (WAT) browning. It also increased the expression of PGC-1α, which is a co-activator of the PPAR/RXR complex, and stimulated the SIRT1-dependent deacetylation of PPARγ and the transcription factor PRDM-16, facilitating PPARγ-PRDM-16 interactions to induce WAT browning [48].

## 2. The ECS and Gastrointestinal Physiology

**Figure 4:**
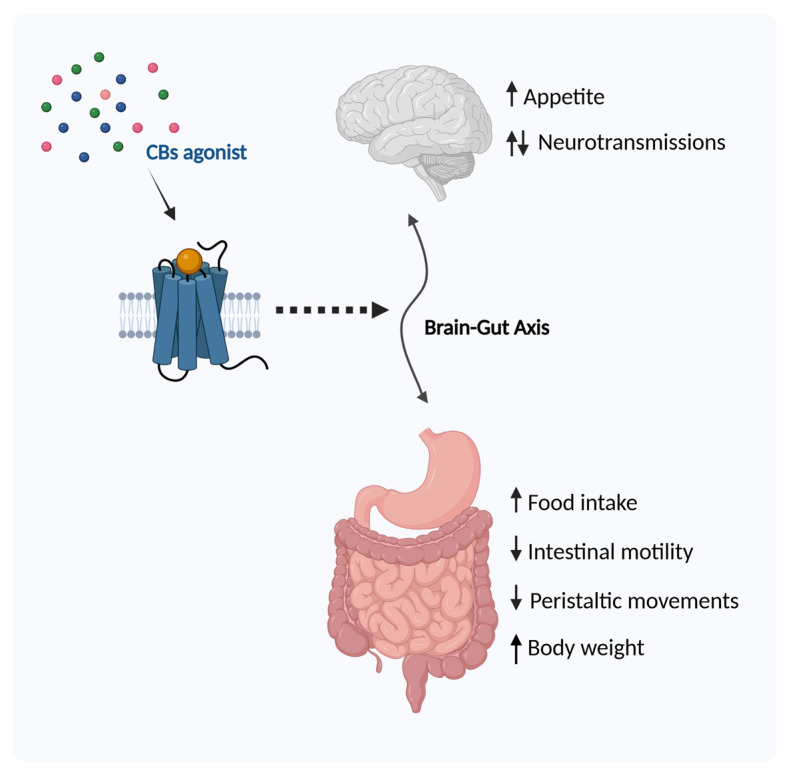
Impact of the ECS on the brain–gut axis. Abbreviation: CBs: cannabinoids.

An increasing number of studies indicate that the endocannabinoid system plays a key role in gastrointestinal (GI) tract function, corroborating the brain–gut connection. CB1Rs are located in the enteric nervous system and in the sensory terminals of vagal and spinal neurons and regulate the release of neurotransmitters, while CB2Rs are mostly distributed in the immune system, indicating their effect on situations of GI tract inflammation [49]. Normal gut physiology is, therefore, influenced by the regulation of the ECS (Figure 4).

### 2.1. Regulation of Food Intake

The ECS has impacts on metabolism, most predominantly anabolic, stimulating protein synthesis, glycogen synthesis, and fat storage. This system, in fact, acts as an appetite regulator, so it is linked to anabolic processes [50,51]. Several studies have demonstrated this regulation. Early studies showed that Δ^9^-THC facilitates food intake during the electrical stimulation of the lateral hypothalamus of Lewis rats [52]. Subsequently, it was shown that the administration of eCB triggered increased food intake. Williams et al. administered AEA systemically to satiated rats. AEA increased their food intake, induced hyperphagia, and modified normal motivational processes. This effect was due to the interaction of AEA with CB1R, whereas by administering SR141716, a selective receptor antagonist, the hyperphagic effect was attenuated [53]. The direct administration of AEA into the ventromedial hypothalamus of satiated rats elicited the same effect as peripheral administration [54]. Kirkham et al. discovered that 2-AG also stimulated feeding in a dose-dependent manner by acting on CB1R, an effect that was attenuated by SR141716A. They also observed that acute food restriction for 24 h resulted in significant increases in AEA and 2-AG levels in the brains of the models and that there was a reduction in hypothalamic 2-AG when the animals were fed an appetizing diet. 2-AG is, therefore, sensitive to changes during feeding, and this observation supports the role of eCBs in appetite control [55]. This result was confirmed by Hanus et al., who hypothesized that if the observations made on dietary restriction in mice are parallel to the human condition, it may be expected that self-imposed dietary restriction in humans, such as in anorexia, may cause a decrease in 2-AG levels, leading to a further reduction in food consumption, thus perpetuating the clinical condition [56]. An opposite effect to AEA and 2-AG was exerted by oleylethanolamide (OEA), which, when administered to freely fed Wistar rats, resulted in an inhibition of appetite that continued for more than 24 h [57].

Thus, AEA and 2-AG may play a role in the regulation of feeding behavior, whereby the latter may act to initiate food intake and the second maintain intake beyond physiological needs.

The effect on food intake correlates with weight gain, so it is interesting to investigate the effect of ECS regulation on obesity.

A randomized study on healthy volunteer patients investigated the effects of dronabinol (DRO), a non-selective cannabinoid agonist, on gastric emptying, small intestine and colon transit, gastric volume, satiety, and postprandial symptoms.

Dronabinol significantly slowed the gastric emptying of a standard solid and liquid meal, particularly in women. However, no significant delay in the small intestine or colon transit was observed in response to the agonist [58].

### 2.2. Obesity

As previously described, endocannabinoids and their receptors have been found, in addition to the CNS, at the peripheral level, as well as in the tissues responsible for regulating energy homeostasis, i.e., the gut [6,59], liver [16], pancreas, and adipose tissue [44]. In light of the involvement of the ECS in the regulation of food intake, energy metabolism, and storage, its role in body weight gain has been explored. Endocannabinoids can be detected in the blood, and the measurement of circulating levels of these molecules has become a prevalent strategy for studying the endocannabinoid system in humans. A recent study analyzed circulating AEA and 2-AG levels in samples from obese female patients (OB), with and without eating disorders, compared to levels in patients with anorexia nervosa (AN) and healthy patients (HC) [60]. They found higher concentrations of AEA in the OB group than in the HC group and AN group, as well as higher concentrations of 2-AG in the HC group than in the OB group with eating disorders. Moreover, AEA was differentially correlated with emotional dysregulation, general psychopathology, food addiction, and body mass index (BMI) in all clinical groups, supporting interactions between biological and clinical factors.

Another group studied the circulating levels of endocannabinoids and the expression of CB1R and the main endocannabinoid degradation enzyme, FAAH, in the adipose tissue of lean and obese women, and after 5% weight loss in a second group of women. They found higher levels of AEA and 2-AG in obese subjects than in healthy subjects; on the other hand, there was a downregulation in the expression of CB1R and FAAH in adipose tissue, suggesting a strong negative correlation between FAAH expression in adipose tissue and circulating endocannabinoids. However, these parameters were not affected by weight loss [61].

Subsequent studies showed that, in obese subjects, increased plasma 2-AG levels were significantly correlated with markers of metabolic syndrome, such as free fatty acids, triglycerides, HDL cholesterol, and adiponectin, confirming the role of endocannabinoids in this syndrome [62,63]. Massa et al. studied the role of the hippocampus and dietary regulation in mice with diet-induced obesity (DIO) by evaluating whether DIO altered the functioning of the hippocampal ECS. Levels of 2-AG and AEA were increased in the hippocampus of DIO mice, demonstrating that DIO-induced changes in the ECS affect not only the tissues directly involved in metabolic regulation but also brain regions that mediate hedonistic aspects of food intake and influence cognitive processes [64].

To elucidate the modulation of the ECS in relation to obesity, several studies have employed the antagonism of CB1R, which seems to be the main receptor involved in this regulation. The main mechanism studied is based on the use of rimonabant, a selective CB1R antagonist. The first study (RIO-Europe) showed the effects of rimonabant treatment when administered for 1 year to obese subjects, verifying significant weight loss, a substantial reduction in waist circumference, and improvements in cardiovascular and metabolic risk factors [65]. Subsequent studies confirmed these drug effects and also demonstrated improvements in glucose homeostasis and insulin sensitivity [66,67]. However, the research was stopped because this treatment caused excessive adverse effects, including mood disorders, depression, anxiety, nausea, and dizziness.

Thereafter, approaches involving new CB1R antagonists with limited or no ability to cross the blood–brain barrier have been tested. Among these, the peripherally active neutral antagonist, AM6545, has been shown to dose-dependently inhibit short-term food intake and reduce body weight gain in rats without inducing adverse reactions. AM6545 blocks the peripheral CB1R binding site by preventing stimulation by the agonist. Employing 7-day chronic treatment with AM6545, body weight gain was inhibited after 4 days, and this effect persisted until the end of the study, but there was no significant inhibition in food intake after day 5, suggesting that the inhibitory actions of AM6545 on body weight gain may not only be caused by inhibiting the food intake [68].

Other positive effects were demonstrated by treatment with the CB1R reverse agonist, JD5037. In mice with DIO, JD5037 was effective in reducing appetite, body weight, hepatic steatosis, and insulin resistance. The key finding of this study is the greater effectiveness of CB1R peripheral reverse agonism, compared with neutral antagonism, in reducing food intake and body weight. In addition, the effects of JD5037 were absent in leptin-deficient *ob*/*ob* and leptin receptor-deficient *db*/*db* mice and in DIO mice treated with a leptin antagonist, indicating the role of leptin on these effects [69]. These findings suggest that the hypophagic effect is mediated by the reverse agonism or antagonism of peripheral CB1R rather than through a central action.

Other therapeutic approaches have been explored involving modifications of the CB1R gene. An early study demonstrated that CB1R knockout mice (CB1R −/−) are lean and resistant to DIO. Both CB1R −/− and wild-type mice were subjected to two feeding regimens: a standard diet and a free-choice diet, offering both standard laboratory food and a high-fat diet (HFD). Under the standard diet, CB1R −/− mice remained lean, while in the free-choice paradigm, their preference for palatable, fat-rich foods was slightly delayed but maintained. Despite the HFD, CB1R −/− mice did not develop obesity [70]. Subsequent studies confirmed these findings and further elucidated the mechanisms through which the ECS regulates energy balance [71,72].

The role of CB2R in obesity regulation has also been explored. The administration of JWH-015, a CB2R agonist, led to a significant reduction in body weight following a temporary decrease in food intake in DIO mice. JWH-015 treatment in these mice also increased the expression of the anti-inflammatory cytokine IL-10 and decreased the expression of the pro-inflammatory marker TNF-α in white adipose tissue, thereby improving obesity-related inflammation [73].

Another study showed that a polymorphism in the gene encoding CB2R, Q63R, which leads to a reduced receptor function, has been linked to eating disorders in humans [74]. Unlike the result with CB1R, genetic deletion of CB2R (CB2R −/−) in mice resulted in an increased food intake and body weight compared to wild-type mice. Additionally, 2-month-old CB2R −/− mice on HFD exhibited reduced weight gain and maintained normal insulin sensitivity, while 12-month-old obese CB2R −/− mice did not develop insulin resistance and showed an enhanced insulin-stimulated glucose uptake. These findings suggest that CB2R plays a role in both age-related and diet-induced insulin sensitivity [75].

Schmitz et al. confirmed the role of CB2R in inflammatory obesity, demonstrating that CB2R deficiency leads to age-dependent obesity in mice, characterized by visceral fat hypertrophy and pro-inflammatory polarization of immune cells in adipose tissue and the liver [76].

A more recent study investigated the modulation of childhood obesity by CB2R, focusing on the CB2R-Q63R variant. The findings suggest that CB2R could be a potential pharmacological target to reduce obesity-related inflammation and excessive fat accumulation in cytoplasmic lipid droplets of WAT, further confirming the interactions between metabolic and immune systems in the pathogenesis of obesity [77]. Given the role of the ECS in both the hedonic and metabolic aspects of eating behavior and body weight, our research group sought to investigate the correlation between BMI and the frequency of the biallelic silent intragenic polymorphism (1359G/A) of CB1R. Our findings revealed a significant association between this CB1R polymorphism and a lower BMI. Therefore, variants of the CB1R gene could be valuable in identifying individuals predisposed to obesity and may influence their responsiveness to endocannabinoids or anti-obesity drugs [78].

A recent clinical study was conducted involving patients with high-risk obesity who were treated with a new CB1R inverse agonist, INV-202 (25 mg per day), for 28 days to evaluate its clinical safety, tolerability, and pharmacokinetic and pharmacodynamic profile.

INV-202 was well tolerated, with no serious adverse effects, treatment discontinuations, or changes in liver or kidney function. The majority (91.6%) of adverse effects were mild in severity. The most common effects were related to the known impact of the CB1R blockade on the gastrointestinal tract, including nausea, diarrhea, vomiting, and decreased appetite. No safety concerns were observed regarding vital signs, electrocardiograms, or physical examinations. By the end of this study, all liver chemistry parameters were within the normal limits for all participants. Additionally, participants treated with INV-202 showed a downward trend in average body weight and waist circumference from baseline to the end of treatment. Significant reductions were observed in body weight, waist circumference, and BMI, along with improving trends in lipid parameters, including total cholesterol, LDL cholesterol, non-HDL cholesterol, and triglycerides [79].

### 2.3. Intestinal Motility

Endogenous and synthetic cannabinoids, particularly AEA, have been shown to act on the small intestine to regulate its motility. AEA, binding to CB1R, has been shown to inhibit electrically induced contractions in the guinea pig myenteric plexus; this effect is enhanced by phenylmethylsulphonyl fluoride, an inhibitor of AEA hydrolysis [80]. Cannabinoid receptor agonists inhibit electrically evoked contractions of the myenteric plexus of the guinea pig small intestine, probably by reducing acetylcholine release through the activation of prejunctional CB1R [81]. Conversely, AEA increases basal acetylcholine release from the myenteric plexus via the stimulation of TRPV1 [82]. Pinto et al. also demonstrated that the non-selective cannabinoid agonists cannabinol, AEA, and WIN 55,212-2 inhibited colonic propulsion in mice in vivo. This anti-propulsive effect is most likely mediated only by CB1R because the inhibitory action is counteracted by the selective CB1R antagonist, SR141716A, whereas the CB2R antagonist SR144528 does not modify the effect of WIN 55,212-2. Furthermore, immunohistochemical data indicate that CB1R immunoreactivity is associated with myenteric and submucosal neurons and fibers in the murine colon [83]. The same group later demonstrated that the main regulator of this inhibitory effect is probably the enzyme FAAH, which catalyzes the hydrolysis of cannabinoids, thus regulating motility [84]. The potential of FAAH in this regard was also confirmed by Salaga et al., who used the inhibitor PF-3845, inducing a potent inhibitory effect on colonic smooth muscle contractility in vitro and on the entire gastrointestinal and colonic transit in vivo. They observed that the regulation of FAAH activity by a potent and selective inhibitor plays an important role in intestinal motility and pain signaling under physiological and pathophysiological conditions [85]. Thus, pharmacological therapies with FAAH-blocking agents, devoid of the undesirable psychotropic effects typical of direct CB1R activation, could offer a new approach to the treatment of intestinal hyperactivity disorders. Grider et al. used a three-compartment preparation of the rat colon to examine the effect of a selective cannabinoid receptor agonist and antagonists on each of the peristaltic reflex tracts: the ascending excitatory, descending inhibitory, and sensory tracts. They show that in each of these tracts, motility is inhibited by endocannabinoids and that the effect is mediated by CB1R and reversed by the CB1R antagonist but not by CB2R or TRPV1 antagonists. Thus, in addition to the already-known action on cholinergic motor neurons, cannabinoids are potent inhibitors of the descending and sensory relaxation components [86]. The role of CB2R on intestinal motility was also revealed. Mathison et al. stimulated increased intestinal transit in rats via the intraperitoneal injection of lipopolysaccharide, which stimulates the development of an inflammatory state. Then, by administering the CB2R agonist JWH-133, the stimulated gastrointestinal transit returned to control values. JWH-133, thus, inhibited the LPS-stimulated gastrointestinal transit in a dose-dependent and significant manner. Finally, they showed that this regulation is mediated by cyclooxygenase, IL-1, and nitric oxide [87]. Human studies have also been conducted in this field, showing that the use of a selective agonist activated CB1R and reduced intestinal motility induced in the colon [88].

A disorder of intestinal motility is gastroparesis, characterized by the delayed gastric emptying of solids (GESs), the absence of mechanical obstruction, and gastrointestinal symptoms such as early satiety, postprandial fullness, nausea, vomiting, bloating, and upper abdominal pain. This condition is most commonly idiopathic or secondary to diabetes mellitus.

Given the known effects of endocannabinoid receptor regulation on visceral sensation and inflammation, a recent study evaluated the effects of 4 weeks of treatment with CBD versus a placebo in patients with non-surgical idiopathic or diabetic gastroparesis. The study assessed symptom response, pharmacodynamics, and safety endpoints. The findings demonstrated that CBD reduced symptoms in patients with idiopathic or diabetic gastroparesis, including a decreased inability to finish a normal meal, fewer vomiting episodes, and reduced abdominal pain. It has been hypothesized that the beneficial effects of CBD may be due to its action on sensory mechanisms or anti-inflammatory effects mediated through CBR2, which may counteract hypersensitivity and the intrinsic inflammatory pathogenesis observed in gastroparesis. Although CBD significantly reduced GES parameters, patients treated with CBD tolerated a greater maximum volume during a nutrient drink test [89].

## 3. ECS and Gastrointestinal Pathology

**Figure 5:**
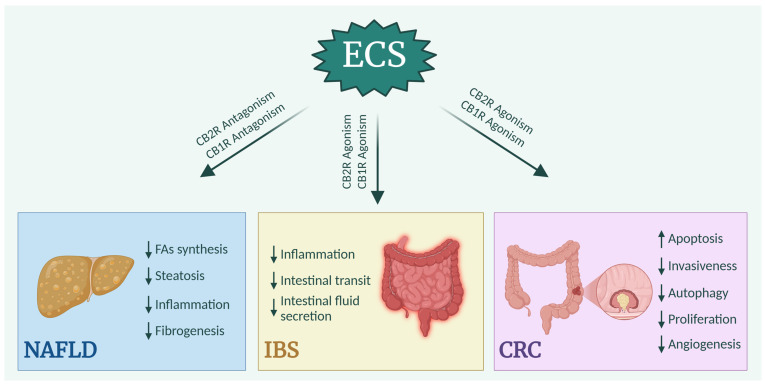
The control of gastrointestinal pathology by CB1R and CB2R. Abbreviations: ECS: endocannabinoid system; CB1R: cannabinoid receptor type 1; CB2R: cannabinoid receptor type 2; NAFLD: non-alcoholic fatty liver disease; FAs: fatty acids; IBS: irritable bowel syndrome; CRC: colorectal cancer.

Even under pathological conditions, the regulation of the ECS has an impact. Understanding these regulatory aspects could, therefore, lead to the development of therapeutic approaches for various gastrointestinal diseases, such as NAFLD, IBS, or CRC (Figure 5).

### 3.1. Non-Alcoholic Fatty Liver Disease

Non-alcoholic fatty liver disease (NAFLD) is a chronic liver disease characterized by hepatic steatosis in the absence of secondary causes such as significant alcohol consumption, steatogenic drug use, or genetic disorders. NAFLD is commonly associated with metabolic risk factors, including obesity, diabetes mellitus, and dyslipidemia. Histologically, NAFLD is classified into two types: a non-alcoholic fatty liver (NAFL), which features hepatic steatosis without hepatocellular damage, and non-alcoholic steatohepatitis (NASH), which is marked by inflammation and hepatocyte damage, with or without fibrosis [90].

The previously described findings illustrate how the ECS, through AEA and 2-AG, increases appetite and promotes fat accumulation, which is a key factor in the development of NAFLD. This suggests that ECS activation may contribute to the progression of liver disease. Considering the rimonabant effects on obesity, Gary-Bobo et al. investigated its impact on hepatic steatosis and the related metabolic syndrome traits (inflammation, dyslipidemia, and low plasma adiponectin levels) in obese Zucker rats. They demonstrated that rimonabant treatment reduced hepatomegaly, fully reversed hepatic steatosis, and lowered plasma levels of liver damage markers (ALT, GGT, and ALP). Additionally, it significantly reduced elevated levels of hepatic TNF-α, a marker of inflammation [91]. Antagonizing CB1R exerts a protective effect against hepatic steatosis and related inflammation. The lipogenic role of hepatic CB1R was demonstrated in CB1R −/− mice, which exhibited a reduced capacity for fatty acid synthesis, further diminished by the CB1R antagonist SR141716. CB1R influences liver lipid metabolism through various mechanisms. CB1R activation was found to induce the expression of the lipogenic transcription factor SREBP-1c and its target enzymes acetyl-CoA carboxylase (ACC1) and fatty acid synthase (FAS), which are key regulators of fatty acid synthesis. The same study showed that obesity induced by HFD significantly increased basal rates of de novo fatty acid synthesis compared to rates in lean controls, which is an effect that was notably reduced by SR141716 pretreatment. In contrast, CB1R −/− mice did not experience changes in basal fatty acid synthesis under the HFD. This outcome was attributed to elevated hepatic AEA levels in animals on the HFD or CB1R −/−, while 2-AG levels remained unchanged [15].

Thus, it can be deduced that peripheral CB1R hyperactivation promotes obesity-associated fatty liver, suggesting that the CB1R blockade could serve as a therapeutic strategy for NAFLD.

In this context, AM6545, a peripheral CB1R antagonist, has been studied in models of obesity-associated NAFLD, demonstrating a reversal of steatosis by improving liver and adipose tissue metabolism [92]. De Gottardi et al. developed an in vitro fatty liver model using immortalized human hepatocytes treated with oleic acid to induce lipid accumulation. When this model was treated with selective CB1R and CB2R agonists, a dose-dependent increase in hepatocyte steatosis was observed, along with an elevated expression of FAS, which is a key enzyme in lipid synthesis. These findings suggest that both CB1R and CB2R play a role in regulating lipid metabolism. Furthermore, the CB2R agonist used in this study enhanced CB1R expression in hepatocytes compared to oleic acid treatment alone, indicating a CB2R-induced cross-regulation of CB1R, which further amplifies the effects of CB1R agonism [93].

Thus, both CB1R and CB2R play a role in maintaining the steatosis state in hepatocytes. Analyzing human liver biopsies, the expression of CB2R was discovered in hepatocytes from patients with NAFLD but not in healthy hepatocytes. A comparative analysis of liver biopsies from healthy individuals, patients with NAFLD, and patients with NASH without significant fibrosis revealed that samples from those with steatosis and non-alcoholic steatohepatitis exhibited immunoreactivity to anti-CB2R antibodies in hepatocytes, cholangiocytes, and hepatic stellate cells. In contrast, no such reactivity was observed in healthy tissue samples [20].

In the context of NAFLD, an inflammatory state develops in the liver. Given the role of CB2R in regulating inflammation, it was hypothesized that this receptor may contribute to disease progression. Early studies showed that treating HFD-fed mice with a CB2R agonist worsened hepatic inflammation and steatosis. Conversely, the knockdown of the CB2R gene in these mice led to a reduced production of pro-inflammatory cytokines and decreased hepatic steatosis, which also contributed to improved insulin sensitivity [94]. This finding supports the involvement of CB2R in the progression to NASH. CB2R appears to have a dual role in the liver, as its expression has been shown to have a positive correlation with both pro-inflammatory cytokine gene expression and the anti-inflammatory protein adiponectin. Additionally, studies investigating the role of CB2R in regulating hepatic fatty acid metabolism in NAFLD patients revealed a positive correlation between hepatic CB2R gene expression and that of ACC1, which is a key enzyme in fatty acid synthesis [15].

Studies on serum samples from NAFLD patients have shown an elevated expression of endocannabinoids in this condition, independent of BMI and obesity. A significant correlation was found between circulating 2-AG levels and triglycerides, as well as several metabolic and hepatic parameters, suggesting the potential role of endocannabinoids in regulating hepatic triglyceride synthesis and release and the development of NAFLD. Conversely, AEA levels did not appear to be elevated in human NAFLD [95].

Additional evidence suggests that the ECS may play a role in the development of human NAFLD by affecting hepatic lipid metabolism. Elevated circulating levels of 2-AG increased the splanchnic production of triglycerides containing saturated fatty acids, and a negative correlation between hepatic CB1R gene expression and liver fat content was observed in individuals with impaired liver function [96].

A key clinical feature of NAFLD patients is liver fibrosis. The role of CB1R in hepatic fibrogenesis was examined using an acute wound repair model induced by the intraperitoneal injection of carbon tetrachloride (CCl_4_). The study assessed the impact of the pharmacological and genetic inactivation of CB1R on the expression of fibrogenic markers, TGF-β1 and α-SMA. Both approaches reduced the expression of these markers and similarly diminished the wound-healing response. Comparable results were also observed in models using different fibrotic agents, such as chronic thioacetamide intoxication and bile duct ligation [97].

The same research group also explored the role of CB2R in fibrogenesis. They initially demonstrated that CB2R is expressed in the liver of subjects with chronic liver disease, specifically in nonparenchymal and biliary cells within and around fibrotic septa, as well as in cultured hepatic myofibroblasts and activated hepatic stellate cells, which are involved in hepatic fibrogenesis in vivo. The activation of CB2R revealed its antifibrogenic properties, leading to dose-dependent growth inhibition and apoptosis of the cells; these effects were reversed by receptor antagonists. The molecular mechanisms underlying these effects in myofibroblasts involve COX-2 induction for growth inhibition and oxidative stress for apoptosis. Supporting this concept, CB2R −/− mice exhibited increased fibrosis, mirroring the in vitro findings observed in hepatic myofibroblasts [21].

Liver injury leads to an inflammatory response by neutrophils, resulting in a phenomenon known as NETosis. This process is characterized by the formation of neutrophil extracellular traps (NETs), the release of granular myeloperoxidase enzymes from lysosomes, and an increase in reactive oxygen species (ROS).

A research group investigated the involvement of CB1R and CB2R in neutrophil chemotaxis during liver inflammation in mouse models of CCl_4_-induced injury. In the damaged liver of these models, a positive correlation was found between CB1R and CB2R mRNA levels and the levels of the neutrophil marker Ly6G. The activation of CB1R, but not CB2R, with an agonist dose-dependently increased neutrophil chemotaxis and NETosis in vitro. Conversely, in CCl_4_-induced murine models, CB1R antagonism reduced Ly6G mRNA levels, neutrophil infiltration, and NET formation. Finally, CB1R-mediated neutrophil activation was shown to depend on p38 MAPK and ROS signaling downstream of Gα_i/o_ [98].

### 3.2. Irritable Bowel Syndrome

Irritable bowel syndrome (IBS) is a functional bowel disorder characterized by irregular bowel habits (such as constipation, diarrhea, or the alternation of these disorders), abdominal pain, impaired gastrointestinal motility, and an altered brain–gut axis, occurring in the absence of any other disease that could cause these symptoms [99,100]. This syndrome is classified into three main subtypes based on the following predominant clinical manifestations: IBS-D, characterized by predominant diarrhea; IBS-C, characterized by predominant constipation; and IBS-M, featuring alternations of diarrhea and constipation.

Enteric expressions of CB1R [101], CB2R [102], and PPARs [28] have been extensively documented with the aim of unraveling the downstream regulatory roles of activation of these receptors. As previously discussed, receptors of the ECS play functional roles in the regulation of intestinal motility. Several studies show that in IBS cases, intestinal motility is altered. Intestinal motor disorders vary in different subtypes of patients [103]. In patients with IBS-D, transit in the small intestine and colon is more rapid than in healthy subjects. This accelerated transit is characterized by a significant increase in the number of high-amplitude propagation contractions (HAPCs). These contractions are strong muscle waves that help move contents through the intestines, and their increased frequency or intensity may contribute to the rapid movement of intestinal contents, leading to diarrhea and other symptoms typical of IBS-D [104]. By contrast, in patients with IBS-C, intestinal transit is slower compared to healthy individuals. This delayed transit is associated with a decreased amplitude of contractions and reduced overall motility within the intestines. These weaker contractions and less frequent movements slow the passage of stools, contributing to the characteristic constipation symptoms seen in IBS-C [105,106,107].

Early evidence suggests that inducing an inflammatory insult in mice with the genetic ablation of CB1R (CB1R −/−) increases their sensitivity to inflammation, indicating that CB1R plays a protective role during inflammatory processes. Similarly, wild-type mice treated with a CB1R antagonist and subjected to an inflammatory insult also exhibit a heightened sensitivity to inflammation, further supporting the protective function of CB1Rs in inflammatory responses [108]. In an inflamed colon state, the increased concentration of the endocannabinoid AEA was observed. This suggests that the upregulation of AEA, an agonist of several endocannabinoid receptors, may exert protective effects by acting not only on CB1R but also on CB2R and TRPV1, both of which are expressed in the colon [109]. The intraperitoneal administration of AEA to mouse models strongly reduced intestinal transit and plasma glucose levels by acting through both CB1R and CB2R [110].

A randomized study in healthy patients given either a placebo or the non-selective CBR agonist dronabinol demonstrated the ability of the ECS to modulate colonic motility. Dronabinol has been associated with colonic relaxation and the inhibition of postprandial increases in tone. Therefore, the stimulation of these receptors could offer a potential therapeutic approach for patients with intestinal disorders characterized by increased motility, such as those with IBS-D [111]. A pharmacogenetic study revealed that the efficacy of dronabinol treatment varies depending on genetic variations in the CB1R gene, and the CNR1 rs806378 CT/TT genotypes show a more delayed colonic transit compared to other genotypes [112,113].

Conversely, IBS-C patients could benefit from therapy by employing a CB1R antagonist, which enhances gastrointestinal motility. In fact, the administration of the CB1R antagonist SR141716A in a mouse model demonstrated that CB1R blockade can accelerate gastrointestinal transit and increase intestinal fluid secretion, thereby promoting evacuation [114]. The administration of taranabant, a CB1R inverse agonist, also alleviated IBS-C symptoms by stimulating intestinal contractility in vitro and gastrointestinal motility in vivo in a dose-dependent manner. This therapy also demonstrated positive effects on abdominal pain, a common symptom in IBS patients, without causing neuropsychiatric side effects [115].

To assess this regulation in humans, the impact of the natural single nucleotide polymorphism (C385A) in the human FAAH gene, which reduces enzyme expression and consequently modulates CB1R, was investigated in relation to the onset of functional gastrointestinal disorders. A significant association between this polymorphism and the symptoms of IBS-M and IBS-D was demonstrated. This genetic variation, which modulates CB1R, regulates smooth muscle contractions and colonic tone, contributing to IBS symptoms [116].

A pilot study reported that FAAH expression differs in patients depending on the subtype of IBS. In IBS-C, the lower expression of the enzyme was found to be correlated with the reduced 2-AG and increased OEA concentration found in these patients. In contrast, FAAH mRNA levels were higher in patients with IBS-D, resulting in higher 2-AG and lower OEA concentrations [117]. Previous studies confirmed the role of palmitoylethanolamide (PEA) and OEA in reducing intestinal motility [118,119].

Regarding the role of CB2R in intestinal motility, it has been shown that CB2R acts under pathological conditions. Tissue preparations from rats treated with LPS demonstrated increased intestinal contractility. This condition was reversed by the activation of CB2R using an agonist, which improved the contractile activity [102]. Similar findings were observed when LPS was administered intravenously in rats, assessing gastrointestinal transit in vivo. Basal transit was influenced only by CB1R agonists or antagonists and not by CB2R. In contrast, LPS-accelerated transit was unaffected by CB1R agonists or antagonists but was significantly reduced, down to control levels, by the CB2R agonist JWH-133. This inhibition was entirely blocked by the CB2R antagonist AM-630. The same study demonstrated that CB2R regulates intestinal transit through a cyclooxygenase-dependent mechanism [66]. These results support the regulatory role of CB2R during inflammation within the gastrointestinal context; in fact, this receptor is the primary regulator of endotoxic inflammation.

A functional role of TRPV1 in this syndrome was also identified. Using biopsy samples from the human colon, an increase in mucosal nerve fibers immunoreactive to TRPV1 was demonstrated in patients with IBS, with no differences between the various subgroups. It is likely that the increase in TRPV1 is a result of the inflammatory state present in these cases and contributes to the pathophysiology of pain in IBS [120]. Further studies on the patient’s rectal biopsy samples revealed that, in cases of IBS, there is a sensitization of TRPV1, probably mediated by the histamine H1 receptor, which is the cause of visceral hypersensitivity and symptoms of this syndrome [121].

In vitro studies on inflammation-induced human Caco-2 cells have shown how OEA and PEA, compounds similar to endocannabinoids, act on TRPV1 and PPARα, respectively, reversing the hyperpermeability associated with inflammatory conditions [122].

Indeed, antagonizing TRPV1 could be a correct approach to reduce inflammation and subsequent visceral colonic hypersensitivity in such patients [123].

A study conducted in our laboratory demonstrated the influence of the endocannabinoid system on the development of IBS. Newborn Wistar rats subjected to stress were fed a ketogenic diet (KD) to assess whether this dietary approach could improve IBS symptoms and gastrointestinal function by examining potential changes in the expression of endocannabinoid receptors in intestinal tissue. After ten weeks of KD treatment, a significant increase in both the gene and protein levels of CB1R and CB2R was observed compared to the control group and the group treated with a standard diet. These results support the hypothesis that manipulating the ECS could be a promising approach for treating IBS by regulating inflammation and intestinal permeability [124].

A randomized clinical trial was conducted to evaluate the effects of dietary supplementation with palmitoylethanolamide and polydatin—which is a dietary compound similar to endocannabinoids—in patients with IBS. No significant differences were found in immune factor parameters between healthy individuals and IBS patients. However, a key finding was the significant reduction in the severity of abdominal pain/discomfort following treatment, as well as a notable decrease in pain/discomfort frequency over time.

The safety profile of palmitoylethanolamide/polydatin was comparable to that of the placebo, and the few adverse events experienced as a result of treatment (such as headaches, upper respiratory tract infections, diarrhea, and vomiting) were not severe [125].

In another clinical study, 75 individuals with IBS (35 with IBS-C, 35 with IBS-D, and 5 with IBS-M) were enrolled to compare the effects of DRO with those of a placebo on colon motility and sensation. In all patients, DRO reduced the motility index of the proximal and distal left colon while fasting, and this caused increased colon compliance compared to the placebo group. The greatest effects were recorded in patients suffering from diarrhea (IBS-D and IBS-M). Furthermore, a pharmacogenetic analysis demonstrated that the effects of DRO on colon compliance and motility may be influenced by genetic variations in FAAH and CNR1, showing that the differences in the effects on colon functions of drugs targeting cannabinoid receptors or the metabolism of AEA have a genetic basis. The most common adverse effects (fatigue 23%, hot flashes 19%, headache 13%, dizziness 11%, brain fog 11%, and elevated heart rate 11%) were mild [113].

Exacerbated intestinal inflammation can lead to more severe pathological conditions than IBS, classified as inflammatory bowel diseases (IBD), including ulcerative colitis (UC) and Crohn’s disease (CD). IBD consists of a subset of inflammatory diseases affecting the small intestine and colon. Patients with IBD may present a variety of symptoms that can differ between UC and CD; however, common symptoms include persistent abdominal pain, diarrhea (with or without blood), fatigue, and weight loss [126]. Targeted treatments aimed at reducing inflammation are necessary in such pathological conditions.

The first clinical study on the effect of cannabis on IBD reveals that cannabinoids may provide anti-inflammatory effects and symptomatic benefits. Although remission of the disease was not achieved, 8-week treatment with THC-rich cannabis resulted in a decrease in the Crohn’s disease activity index in 90% of patients without producing significant side effects [127].

The components of the ECS are differentially expressed in human IBD. AEA is reduced in the inflamed mucosa of IBD as a result of decreased NAPE-PLD activity and increased FAAH activity. The expression levels of the main receptors are also altered, with the higher expression of CB1R in inflamed mucosa compared to non-inflamed mucosa in both CD and UC patients. The administration of a non-hydrolyzable AEA analog, methanandamide (MAEA), to mononuclear cells from the lamina propria and biopsies from IBD has shown anti-inflammatory effects both in vitro and ex vivo, negatively regulating the production of the pro-inflammatory cytokines IFN-γ and TNF-α [128].

A clinical study investigated the long-term effects of therapeutic cannabis in patients with IBD. It was found that the most effective dose of cannabis was 30 g/month of raw cannabis, or 21 mg/day of THC and 170 mg/day of CBD. The use of other medications after 1 year of cannabis consumption was significantly reduced for all treatment options. Additionally, the level of occupation during the year of cannabis use increased, suggesting an improvement in quality of life. Functional improvements were also reported by patients’ relatives, indicating that most patients showed no signs of dependency. The mild side effects described by patients included a dry mouth (63%), memory decline (34%), eye irritation (14%), dizziness (13%), confusion (9%), and restlessness (8%) [129].

A recent clinical study assessed whether 8 weeks of cannabis treatment could affect eCB levels and clinical symptoms in patients with IBD. The levels of PEA, AEA, and AA significantly decreased over time in the placebo group of UC patients, likely due to increased FAAH expression, while cannabis consumption prevented this decrease. The disease severity index (Lichtiger score) and quality of life improved significantly in the treatment group compared to the placebo group, and higher eCB levels were associated with fewer bowel movements. These results suggest that IBD may be associated with an altered “tone” of eCBs [130].

Population-based analyses also confirm that patients with IBD use cannabis for therapeutic purposes to alleviate symptoms [131,132].

Recently, an in vivo study tested the therapeutic effect of *Cannabis sativa* on acetic acid-induced colitis in rats. Animals treated with *C. sativa* at doses of 20 and 40 mg/kg showed a 168% and 400% increase, respectively, in CB1R levels compared to the animals with UC. Additionally, SIRT-1 levels were significantly increased, resulting in a reduction in inflammatory markers such as NF-κB, MAPK, and ERK. Histological analysis also showed improvements from the treatment, with notable beneficial effects on the submucosal mucosa and the superficial epithelium, which were almost normal at the highest dose administered [133].

### 3.3. Colorectal Cancer

Colorectal cancer (CRC) is one of the most common cancers worldwide and a significant cause of morbidity and mortality. It arises in cells in the colon or rectum; most CRCs develop from adenomatous polyps that undergo malignant transformation over time.

In its early stages, colorectal cancer may not exhibit obvious symptoms. However, the most common signs include changes in bowel habits, rectal bleeding, abdominal pain, and weight loss. The primary treatment is usually surgery, particularly when the disease is detected early, while chemotherapy, radiotherapy, or biological therapies are employed in more advanced stages or when metastases have developed [134,135].

The prognosis is generally favorable when the disease is detected early but worsens as metastatic spread increases. In fact, most cancer-related deaths occur in patients with metastases, due to limited treatment options. Metastatic CRC, in particular, has restricted treatment options and consequently high mortality rates [136,137]. In this context, understanding the molecular mechanisms and factors influencing metastasis formation is crucial in developing effective cancer therapies before this stage.

Further investigation into the modulation of molecular mechanisms underlying tumor and metastatic development, using alternative molecules to conventional drugs or those that can effectively complement established therapies, could prove beneficial.

The first evidence of the anti-tumor effects of cannabinoids dates back to the 1970s when Munson et al. demonstrated that Δ^8^-THC, Δ^9^-THC, and cannabinol (CBN) are capable of suppressing tumor growth in vitro and prolonging the survival of a murine model of Lewis lung adenocarcinoma [138].

After the discovery of endocannabinoid receptors in the 1990s, studies were conducted to clarify the role of endocannabinoids in tumor development. The first of these was a study by De Petrocellis et al., which demonstrated that anandamide, a CB1R agonist, inhibited the proliferation of human breast cancer cells in a dose-dependent manner. This effect was mediated by the CB1-like receptor, inhibiting the endogenous action of prolactin at the prolactin receptor level [139]. Subsequent studies have investigated the role of the ECS in cancer therapy using in vitro and in vivo models [140,141,142,143]. The anticancer mechanisms of cannabinoids may involve not only the inhibition of proliferation but also the suppression of tumor angiogenesis [144], induction of apoptosis [140,145], promotion of autophagy [146,147], and cell cycle arrest [148].

In the context of CRC, it has been demonstrated that the activation of cannabinoid receptors can induce apoptosis in cancer cells, inhibit proliferation, and reduce the invasiveness and metastatic potential of neoplastic cells. Treatment with THC led to the CB1R-mediated inhibition of the RAS-MAPK/ERK and PI3K-AKT survival signaling pathways—two critical cellular pathways often dysregulated in colorectal cancer—and the activation of the pro-apoptotic Bcl-2 family member BAD [149]. Endocannabinoid levels have been found to be twice as high in cancerous colon tissue compared to normal mucosa. Furthermore, it has been demonstrated that endocannabinoids have the ability to inhibit the growth of CRC cells in culture. These effects are largely mediated through the stimulation of their receptors [150].

A study showed that the activation of CB1R and CB2R, using their respective agonists, induced apoptosis in colon cancer cells, mediated by the de novo synthesis of ceramide, which acts as a pro-apoptotic second messenger [151].

A genetic and pharmacological study revealed that in 18 out of 19 CRC samples and 9 out of 10 tumor cell lines, CB1R expression was significantly reduced compared to adjacent normal mucosa and healthy cells. This downregulation was associated with the strong promoter methylation of the CB1R gene, suggesting that elevated levels of endogenous cannabinoids might not affect tumor growth because the loss of CB1R in most CRC cases would render the cells resistant to these ligands. Therefore, a combination of two treatment approaches, such as a demethylating agent followed by a CB1R agonist, could prove more effective [152]. The same study also investigated the downstream anti-tumor mechanism of CB1R. Treatment with a CB1R agonist, through a cAMP-dependent PKA pathway, reduced the expression of survivin, an apoptosis inhibitor that is overexpressed in most human cancers.

A potential regulatory role for COX-2 in these cellular aspects was identified. COX-2 is frequently upregulated in colorectal cancers, where it metabolizes the endogenous cannabinoid anandamide into prostaglandin-ethanolamides (PG-EAs). Prostaglandins (PGs) are thought to mediate some of the tumor-promoting effects of COX-2. Therefore, treatment with AEA, in addition to its anti-tumor effects as a CBR agonist, exerts an antiproliferative effect regulated by COX-2. By promoting the production of PGE2-EA from AEA, this process results in growth inhibition and induces cell death [153]. Even in apoptosis-resistant cells, AEA has the same effect through the regulation of COX-2 expression [154].

Recently, the anti-tumor ability of the synthetic cannabinoid URB447, a CB2R agonist, was tested in CRC and melanoma in vitro. The results revealed that treatment with this agonist can reduce the viability of cells of both tumor types in a dose-dependent manner, promoting apoptosis and dysregulating the cell cycle by blocking cells in the G0/G1 phase [155]. This study confirmed previous findings that claimed the ability of CB2R agonists, such as CBD, to reduce CRC cell viability via increasing caspase-3/7 activity together with stimulating poly (ADP-ribose) polymerase (PARP) and inducing reticulum stress [156]. The use of CB2R agonists has also shown that downstream of this signaling, proliferation arrest is due not only to the induction of caspases but also to a significant downregulation of anti-apoptotic Bcl-2 [157].

In vivo studies have also demonstrated that CBD is able to reduce CRC by acting through CB1R. In a murine model of azoxymethane (AOM)-induced colon cancer, CBD was administered at two different doses (1 mg/kg or 5 mg/kg). The greatest efficacy was observed with a lower dose of CBD, which reduced the formation of aberrant crypt foci (ACF)—early neoplastic lesions—as well as polyps and tumors. This effect was attributed to the upregulation of the active fragment of caspase-3, inducing a pro-apoptotic effect in cancer cells. These antiproliferative effects were further confirmed in two different colorectal carcinoma cell lines, Caco-2 and HCT116, through the involvement of CB1R, TRPV1, and PPARγ [158].

Previously, the same research group demonstrated that the administration of an FAAH inhibitor to murine models of AOM-induced colon cancer also reduced the number of ACF through pro-apoptotic effects. Additionally, this treatment increased the levels of 2-AG, which, in turn, exerted protective effects, likely through the action of CB2R [159]. Similar effects were observed following the treatment of CRC cell lines with cannabigerol (CBG), a non-psychotropic cannabinoid.

CBG acts mainly as an antagonist of TRPM8, which is involved in the regulation of cell proliferation/apoptosis. By treating cells with CBG, an antiproliferative effect was obtained based on pro-apoptotic signaling downstream of TRPM8, probably associated with the overproduction of ROS [160].

To assess the safety and tolerability of CBD administration, a Phase I randomized study was conducted, involving either a single ascending dose (1500, 3000, 4500, or 6000 mg) or multiple doses (750 or 1500 mg twice daily for six days, with a single dose on the morning of the seventh day) of orally formulated CBD in healthy subjects.

The analysis showed that even the highest doses administered twice daily were well tolerated, with only mild to moderate adverse effects reported. The most common adverse effects were diarrhea, nausea, headache, and drowsiness. No deaths occurred, no participants discontinued the study due to adverse effects, and no other serious effects were observed [161].

A recent study investigated the role of the ECS in CRC using various murine CRC models (spontaneous cancer, AOM/DSS-induced colorectal cancer, and hereditary Apc ^Min/+^ colon cancer), evaluating the differences between wild-type mice and CB2R knockout mice (CB2R −/−). CB2R −/− mice developed more spontaneous precancerous lesions in the aging model and an increased number of tumors in the chemically or genetically induced CRC model. Both CB2R −/− models with induced CRC showed enhanced immunosuppressive myeloid-derived suppressor cells and reduced anti-tumor CD8+ T cells at the splenic level. These results suggest that the endogenous activation of CB2R suppresses the development of colon cancer by modulating the balance between pro-tumorigenic and anti-tumorigenic immune cells [162].

In our laboratory, several experiments were conducted to investigate the connection between the ECS and the development and progression of CRC. Given the influence of polyamines on cell proliferation and their dysregulation in neoplastic conditions, the effects of AEA treatment on polyamine levels and cell proliferation were examined in three human colon cancer cell lines with different degrees of differentiation and positivity for CB1R. The results demonstrated that the administration of increasing concentrations of AEA led to a significant reduction in both the single and total polyamine content in all the cell lines studied. Furthermore, AEA significantly reduced the proliferation of all tested cell lines, mainly at higher treatment doses. However, the treatment led to a significant increase in CB1R mRNA in only one of three cell lines. All treatment effects were reversed following the administration of SR141716A, a CB1R antagonist. These data allowed us to deduce that AEA may act as an endogenous inhibitor of colon cancer cell growth by binding to CB1R. Additionally, the reduced polyamine levels following this treatment suggest that polyamine metabolism could be a target for the antiproliferative and antineoplastic properties of endocannabinoids [163].

Another study conducted by our group on 59 CRC patients aimed to identify a correlation between CB1R expression and the severity of metastatic disease. For the initial result, a significant decrease in both the gene and protein expression of CB1R was identified in colonic tumor tissue compared to the surrounding normal mucosa, suggesting that the receptor expression was linked to the ongoing neoplastic process. Additionally, a significant downregulation of CB1R gene expression was observed in patients with synchronous metastases, both in the normal mucosa and in the tumor tissue. To clarify the consequences of this dysregulation, we investigated the levels of effector proteins downstream of CB1R. Intracellular levels of p38 MAPK were significantly reduced in both the normal mucosa and tumor tissue of patients with synchronous metastases, as well as in the tumor tissue of patients without metastases. Furthermore, activation levels of ERK 1/2 proteins were found to be lower in tumor tissue than in normal tissue. In contrast, the activation levels of the Akt protein were higher in both tumor and normal tissues in patients with CRC and metastasis compared to those of patients without metastasis. The ability to develop metastases was attributed to lower levels of anti-apoptotic proteins than of pro-apoptotic proteins, a regulation occurring downstream of Akt activation. Thus, a comprehensive analysis of the data led us to conclude that reduced CB1R expression is closely linked to molecular changes induced by the activation or inhibition of downstream signaling pathways associated with this receptor. Since these signaling pathways appear dysregulated even in the normal intestinal mucosa surrounding the neoplasia, it can be inferred that molecular defects initially present in the normal tissue contribute to the development and progression of the tumor [164].

We also tested the ability of CB1R to respond to hormonal stimulation by estrogen. In human colon cancer cells, estrogen treatment exerts a positive influence on CB1R expression levels, resulting in a significant antiproliferative action and increased apoptotic rate in a dose-dependent manner. Therefore, we could deduce that the anti-tumor action of estrogen on CRC may be due to its regulation of the CB1R gene and protein levels [165].

Given the ability of estrogen to stimulate CB1R, we tested the use of quercetin in CRC cell lines. Quercetin is a phytoestrogen and natural flavonoid found in many fruits, vegetables, and tea and has attracted considerable attention because of its anti-tumor properties. Experimental studies have shown that it exerts antiproliferative and antineoplastic effects on various types of cancer cells, including colon cancer. As a phytoestrogen, quercetin can modulate the expression of estrogen-sensitive cell receptors such as CB1R. As expected, quercetin increased CB1R gene expression. To understand how CB1R contributes to the effect of quercetin, we investigated some of the key molecular pathways regulated by this phytoestrogen. We observed that quercetin is able to alter the balance between cell proliferation and apoptosis by blocking the cell cycle and inducing tumor cells to undergo cell death. Analyzing the activation of key factors involved in cell proliferation and apoptosis, we found that proliferative markers such as GSK3β, PI3K, Akt, and STAT3 are reduced, while β-catenin and pro-apoptotic markers such as JNK and c-Jun are increased. Finally, using a scratch test, we observed that quercetin inhibits the in vitro migration of Caco2 cells. All the regulatory effects are enhanced by Met-F-AEA, an AEA analog, and partially reversed by SR141716, a CB1R antagonist, demonstrating quercetin’s ability to bind to the receptor. These findings provide new opportunities for the development of anti-tumor therapeutic strategies based on the combination of quercetin and cannabinoids aimed at activating cell death mechanisms in tumor cells. These effects are due to the synergistic interaction between different signaling pathways activated both directly by quercetin and through the induction of CB1R [166].

In a mouse model of azoxymethane (AOM)-induced CRC, a diet supplemented with quercetin regulated gene expression and the protein levels of CB1R while inhibiting STAT3 and phosphorylated STAT3 (p-STAT3), which are both mediators of cell proliferation. Furthermore, dietary quercetin significantly increased the Bax/Bcl2 protein expression ratio [167].

A clinical study conducted in Israel between 2015 and 2017 examined 2970 cancer patients who received medical cannabis. The study aimed to analyze the patient population and evaluate the safety and effectiveness of this treatment. Among the participants, 7.9% were diagnosed with colorectal cancer. Medical cannabis was administered as palliative care to help manage symptoms such as pain, loss of appetite, fatigue, sleep disturbances, and nausea. The treatment involved four types of cannabis: high-THC sativa strains without CBD, high-THC indica strains without CBD, balanced strains with equal amounts of THC and CBD, and CBD-rich strains.

Pain intensity and quality of life were assessed after six months of treatment. During the follow-up period, 24.9% of patients passed away, while 18.8% discontinued treatment. Among those who continued, 60.6% responded positively to the therapy. Specifically, 95.9% experienced significant improvement, 3.7% reported no change, and 0.3% noted a deterioration in their condition. Before treatment, only 18.7% of patients rated their quality of life as good, whereas after six months, this percentage increased to 69.5%.

The most improved symptoms included pain, nausea, vomiting, sleep disorders, anxiety and depression, itching, and headaches. In terms of safety, the most commonly reported side effects were dizziness, dry mouth, increased appetite, and drowsiness.

Among colorectal cancer patients, the success rate after six months of treatment was 58.3%. These findings suggest that medical cannabis, by alleviating multiple symptoms, represents a valuable therapeutic option for cancer patients [168]. This study confirmed the findings of previous research on cannabinoid-based treatments in cancer patients, demonstrating good tolerance, effective pain relief, and the absence of addiction or severe adverse effects [169], even in long-term treatments [170].

Moreover, in vitro and in vivo studies have validated the properties of cannabinoids and their ability to influence the angiogenic response through VEGF downregulation, the inhibition of epithelial to mesenchymal transition (EMT), and lead to a reduction in metastatic growth [155,171,172,173].

Several forms of evidence have demonstrated that the ECS can also interact with the gut microbiota to regulate intestinal permeability and inflammation. This regulation was first discovered in 2007 through the oral administration of specific *Lactobacillus* strains to mice and rats. These strains were able to induce the expression of CB2R via the NF-κB pathway, contributing to the modulation and restoration of normal visceral pain perception [174].

Subsequently, various models of microbiota manipulation were developed, consistently resulting in alterations in the abundance of CB1R, FAAH, and MAGL mRNA [175,176,177].

Therefore, it can be inferred that microbiota can regulate the intestinal eCB tone, but changes in intestinal eCB tone could also influence microbiota composition. Several studies have shown that alterations in the ECS are reflected in changes at the microbiota level [178,179,180].

Therefore, dysbiosis and the dysregulation of the intestinal ECS may promote altered gut conditions, potentially increasing susceptibility to CRC.

The combination of cannabis-based treatments with interventions targeting the gut microbiome could provide synergistic effects, enhancing the therapeutic properties of cannabinoids.

The interaction between cannabinoids and microbiomes could represent a promising strategy for the prevention and treatment of CRC due to their ability to modulate microbial composition, reduce inflammation, and influence tumor growth. However, further research is needed to better understand these mechanisms and their therapeutic potential.

## 4. Conclusions

This review illustrates the central role of the ECS as a therapeutic target for gastrointestinal and metabolic disorders, offering prospects for the development of more effective and personalized treatments.

The ECS emerges as a crucial regulator of normal gastrointestinal functions, integrating the brain–gut connection. CB1R and CB2R play distinct roles: the former is involved in the control of neurotransmission and intestinal motility, while the latter modulates inflammatory responses. Regarding food intake, endocannabinoids such as AEA and 2-AG have been shown to stimulate appetite through CB1R, whereas antagonists, like OEA, exert the opposite effect by inhibiting food intake. These mechanisms indicate a close relationship between the ECS and metabolic regulation.

Regarding intestinal motility, endocannabinoids inhibit peristaltic movements, primarily through CB1R, by reducing acetylcholine release. Inhibitors of the FAAH enzyme, which degrade endocannabinoids, show therapeutic potential for use in hyperactivity intestinal disorders, providing benefits without psychotropic effects. Additionally, CB2R plays a significant role in modulating motility under inflammatory conditions.

Regarding the pathological aspects of the gastrointestinal system, research has demonstrated that ECS dysregulation is closely linked to comorbidities of obesity, such as metabolic syndrome and NAFLD.

The involvement of CB1R and CB2R in metabolic processes has been extensively studied, highlighting their impact on lipid accumulation, inflammation, and insulin sensitivity. CB1R activation is strongly associated with weight gain and metabolic dysfunction, while its antagonism offers promising therapeutic effects, including appetite reduction, weight loss, and improved metabolic profiles, although the use of central CB1R antagonists has encountered limitations due to adverse effects. CB2R also influences obesity, primarily through its role in modulating inflammation and insulin sensitivity. Genetic studies further underscore the importance of CB1R and CB2R variants in targeting a predisposition to obesity and treatment response.

The ECS has been shown to play a significant role in the pathogenesis and progression of NAFLD, influencing hepatic lipid metabolism, inflammation, and fibrogenesis. CB1R and CB2R, along with endocannabinoids such as AEA and 2-AG, contribute to the regulation of lipid accumulation and inflammatory responses, revealing them as potential therapeutic targets. CB1R hyperactivation is associated with increased hepatic steatosis and fibrogenesis, while its antagonism, both centrally and peripherally, has demonstrated protective effects in preclinical models, including reductions in steatosis, inflammation, and fibrosis. Similarly, CB2R exhibits a dual role, promoting inflammation and steatosis in certain contexts while demonstrating antifibrogenic and anti-inflammatory properties when appropriately modulated.

The ECS also plays a role in intestinal disorders such as IBS. Receptors, along with enzymes and related compounds, contribute to the modulation of motility, inflammation, and visceral hypersensitivity, with subtype-specific (IBS-D, IBS-C, and IBS-M) effects. CB1R activation is primarily protective, regulating motility and inflammation, while CB2R acts mainly under pathological conditions, attenuating inflammatory responses. Additionally, TRPV1 has been implicated in pain mechanisms and visceral hypersensitivity, thus offering another potential therapeutic target. Emerging evidence supports dietary approaches, such as the ketogenic diet, in modifying ECS expression and alleviating IBS symptoms. Therapeutic strategies targeting the ECS offer promising avenues for IBS management.

Furthermore, clinical and preclinical studies suggest that cannabis, particularly cannabinoids like THC and CBD, may offer therapeutic potential by reducing inflammation and improving symptoms in IBD patients. While remission has not yet been achieved in clinical settings, significant improvements in disease activity and quality of life have been reported with cannabis treatment.

Finally, the ECS plays a multifaceted role in the development, progression, and potential treatment of CRC. CB1R and CB2R, activated by their endogenous ligands, influence critical processes such as tumor cell proliferation, apoptosis, angiogenesis, and metastasis. The dysregulation of CB1R expression, often observed in CRC, is linked to tumor progression, while its activation or restoration may offer therapeutic benefits. Similarly, CB2R has demonstrated anti-tumor effects, including the modulation of immune cell dynamics and the promotion of apoptosis in tumor cells. Therapeutic strategies leveraging cannabinoids or cannabinoid-inspired compounds, such as selective agonists or FAAH inhibitors, show promising anti-tumor properties. Additionally, natural agents like quercetin may enhance CB1R expression and synergize with cannabinoids to regulate key signaling pathways, offering potential for novel combination therapies.

Emerging evidence highlights the significant role of microbiota in CRC. The dysregulation of the gut microbiota, along with alterations in the intestinal ECS, may contribute to increased susceptibility to CRC. The interaction between cannabinoids and the microbiome presents a novel approach that could enhance the therapeutic properties of cannabinoids by regulating microbial composition, reducing inflammation, and influencing tumor growth.

In conclusion, these findings suggest that the ECS offers a versatile approach for modulating gastrointestinal physiological aspects and treating conditions such as obesity and its complications, IBS, and CRC. Future research should refine ECS-targeted therapies to maximize their efficacy and minimize adverse effects, unlocking new opportunities for innovative treatments of disordered metabolism, inflammation, and cancer. Clinical studies show that medical cannabis could be a valuable adjunct to cancer and treatments for inflammation, providing symptom relief and improving patients’ overall quality of life. However, further research is needed to refine treatment protocols and explore their full therapeutic potential. 

## Data Availability

The data are contained within this article.
